# Mild Traumatic Brain Injury by the Glasgow Coma Scale Score and Early CT-Brain Findings in Acute Alcohol Intoxication Patients: A Prospective Observational Study

**DOI:** 10.7759/cureus.67669

**Published:** 2024-08-24

**Authors:** Navin S Arunachalam Jeykumar, Gopalakrishnan M Niban, Pandiyan Vadivel, Sumathy Masanam Kasi

**Affiliations:** 1 General Surgery, Thanjavur Medical College, Thanjavur, IND; 2 Neurological Surgery, Thanjavur Medical College, Thanjavur, IND; 3 Medical Education, Jawaharlal Institute of Postgraduate Medical Education and Research, Pondicherry, IND

**Keywords:** glasgow coma scale score, road traffic accidents, mild traumatic brain injury, acute alcohol intoxication, early ct-brain

## Abstract

Introduction

Traumatic brain injuries (TBI) in recent years have proved to be a significant public health problem, with potentially life-changing consequences for the individual and their family. Alcohol consumption is a regular, well-documented problem among persons sustaining TBI due to road traffic accidents and accidental falls. The primary objective of this study was to find out the correlation between the Glasgow Coma Scale (GCS) score and CT brain findings among mild TBI patients under acute alcohol intoxication and determine if early CT-brain is indicated in this group.

Methods

A prospective observational study was conducted involving 111 alcohol-intoxicated patients with mild head injuries admitted to the surgical wards of Thanjavur Medical College Hospital over a period of three months. The Glasgow Coma Scale was used to assess the patient's neurological status and determine the severity of the brain injury. A semi-structured CT-brain findings chart and a severity of alcohol intoxication objective-based scoring system were developed and validated by experts. Descriptive statistics tools such as frequency, percentage, and mean were used, along with inferential statistics tools like the Chi-squared test, Fisher exact test, and Pearson's correlation coefficient test.

Results

The study findings showed that the comparison of GCS with early CT-brain was significant at a p-value of 0.012, and a negative correlation (r=-0.253) was found between GCS and CT-brain findings. A comparison of CT-brain findings with the severity of alcohol intoxication was non-significant at a p-value of 0.433.

Conclusions

Early CT-brain in intoxicated mild TBI patients may have a positive impact on early diagnosis and management, even in centers with limited resources catering to low-income population groups. The results of our short-term study show that early CT-Brain picks up lesions and helps initiate early management while it is up to the attending physician to keep in mind an adverse cost-benefit ratio in overuse of hospital resources and misdiagnosis leading to undertreatment causing long-term sequelae and morbidity before prescribing early CT-brain in this cohort of patients.

## Introduction

TBIs are a significant public health problem with severe, life-altering consequences for individuals and their families. TBIs necessitate multidisciplinary tertiary medical care with substantial economic costs that could be prevented. In India, 1.5 to 2 million people suffer TBIs annually, with 1 million resulting in death [1,2]. Road traffic accidents (RTA) account for 60% of TBIs, 20-25% are caused by falls, and 10% are due to violence [3]. Hospital records show head injury accounts for 3% to 4% of emergency department cases and remains the leading cause of death and disability from childhood to early middle age. While 92% of the world's RTA fatalities happen in low- and middle-income countries, more than 50% of all road traffic deaths are among vulnerable road users, including pedestrians, cyclists, and motorcyclists [4]. Overall, 15-20% of TBI involves alcohol intoxication [3].

The most frequent clinical manifestations in TBI patients are headache, vomiting, and skull fractures, often accompanied by a history of loss of consciousness. Additional clinical features indicative of a basal skull fracture include nosebleeds, ear bleeding, bruising over the mastoid process, and the presence of cerebrospinal fluid otorrhoea and rhinorrhoea [5].

Alcohol intoxication significantly contributes to head injuries and complicates the diagnosis, severity assessment, and management of these injuries [6,7].

Selecting patients for CT scans is a critical triage decision in mild TBI, as it allows for early identification of lesions that may necessitate hospital admission or life-saving surgery, potentially preventing long-term morbidity from secondary brain injury. While moderate head injury patients (Glasgow Coma Scale (GCS) score <13) are required to undergo an early CT-brain (within an hour of admission), there are no clear guidelines for using the GCS to determine the need for a CT brain scan in alcohol-intoxicated patients with mild TBI. Few interventional studies have been conducted in India on this topic. Evidence-based practice is essential for promoting quality care. This study aims to determine the necessity of early CT brain scans in patients with mild TBI under alcohol intoxication in a tertiary care hospital. The objective is to correlate the GCS with CT findings among alcohol-intoxicated patients with mild head injuries.

## Materials and methods

Study design

A prospective observational study was conducted at a single center, Thanjavur Medical College Hospital in Tamil Nadu, India, for three months from the months April to June 2020, with the approval from the Institutional Human Ethics Committee (Approval no: 626).

Participants

The study included adult patients who had sustained mild head injuries due to road traffic accidents (RTA) or accidental falls under the influence of alcohol within 24 hours of the incident. Patients were excluded if they had not consumed alcohol within 24 hours prior to the incident, sustained injuries to other organ systems without head injury, were brought dead, or had moderate (GCS 9 to 13), severe (GCS 5 to 8), or very severe head injuries (GCS <5). The sample size was 111 alcohol-intoxicated mild head injury patients who presented to the emergency department and the general surgery outpatient department.

Firstly, when the patients were brought to the emergency and outpatient departments (N=260), they were objectively examined, and the severity of head injuries was categorized using the Glasgow Coma Scale (GCS). Secondly, the level of alcohol intoxication was determined using an objective-based scoring tool developed at the researchers' hospital. This tool categorized patients based on seven signs of alcohol intoxication: smell of alcohol, slurred speech/increased verbosity, abnormal gait/ataxia, confusion/excitement, amnesia, nystagmus, and bloodshot eyes. The tool used a three-point Likert scale to categorize the intoxication levels as mild (score 0-2), moderate (score 3-5), and severe (score 6 and above).

Subsequently, patients classified as mild head injuries (GCS scores of 13, 14, or 15 with a history of loss of consciousness) and who were intoxicated, regardless of the level of intoxication, underwent early CT-brain scans and additional imaging to check for concomitant injuries. All CT films were reviewed and reported by a radiologist. The complete findings for each patient were recorded using a semi-structured CT-brain findings chart developed by the researcher.

**Figure 1:**
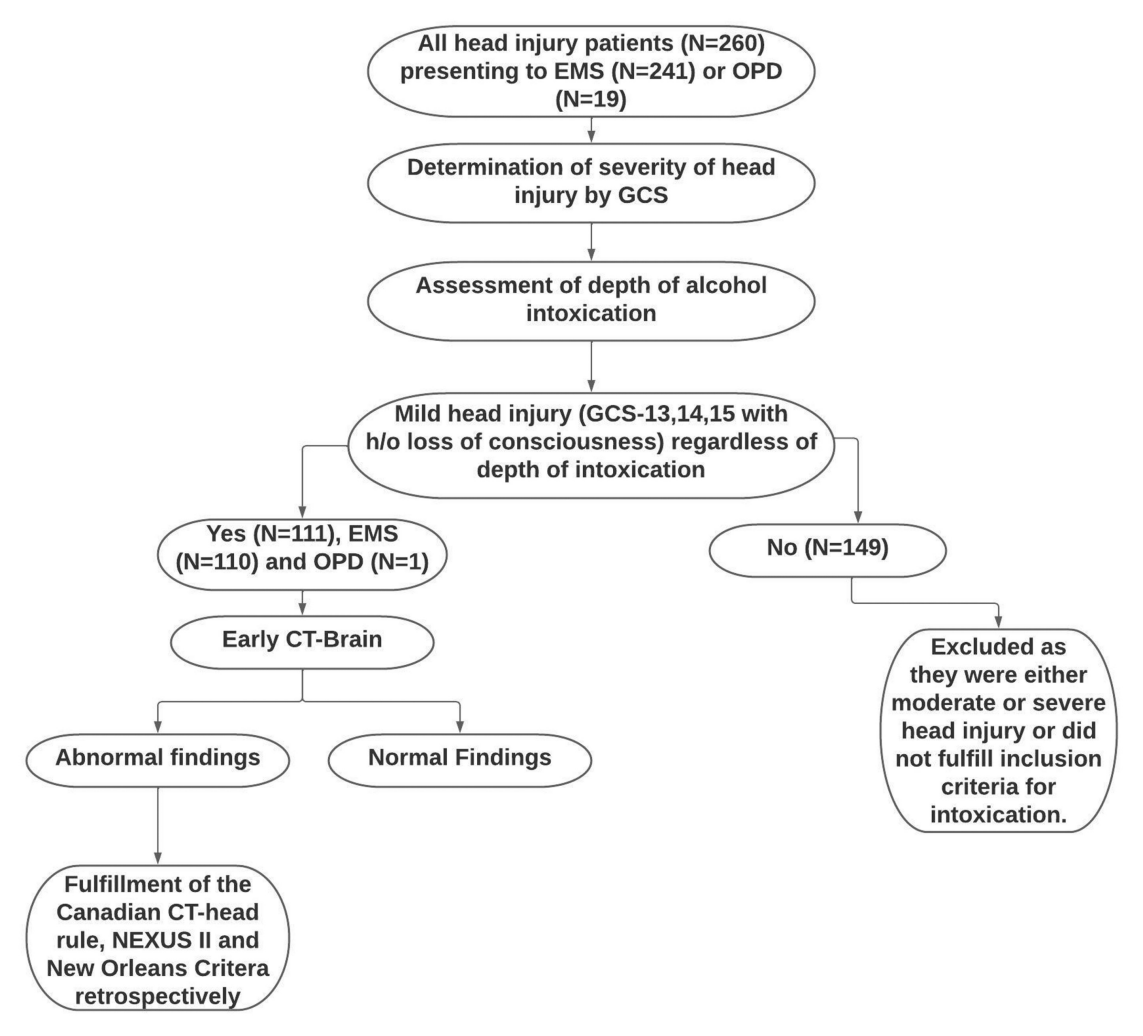
Flowchart outlining the steps of data collection OPD - outpatient department; GCS - Glasgow Coma Scale

Finally, for patients with positive CT findings suggestive of head injury, a retrospective evaluation was conducted to determine how many met the criteria for early CT-brain imaging according to the Canadian CT Head Rule, National Emergency X-Ray Utilization Study II (NEXUS-II), and New Orleans Criteria. Figure 1 illustrates the detailed steps of data collection.

The data was analyzed using SPSS version 20.0 (IBM Inc., Armonk, New York). Descriptive statistics were used to summarize demographic variables, while inferential statistics, including Chi-squared tests, were employed to examine associations between GCS and CT-brain findings, and levels of alcohol intoxication and CT-brain findings to achieve the research objectives.

## Results

Data from 111 patients meeting the inclusion criteria was collected and analyzed using SPSS version 20. Descriptive statistics were utilized to assess the demographic variables, as detailed in Table 1.

**Table 1:** Demographic and clinical characteristics of the study population Data are presented as mean ± standard deviation (SD) and n (%). Total N=111. The mean age was 37.1 years with s standard deviation of 15.2 years. The age range of the participants was between 18 and 75 years. GCS - Glasgow Coma Scale

Characteristics	Mean ± SD / n (%)
Total number of patients	111
Age (years)	37.1 ± 15.2
<30 years	37 (33.3%)
30-39 years	25 (22.5%)
40-49 years	23 (20.7%)
50-59 years	16 (14.4%)
≥60 years	10 (9.0%)
Gender	
Male	111 (100%)
Number of days after which admission occurred	
On same day	97 (87.4%)
After one day	9 (8.1%)
After two days	2 (1.8%)
After three days	1 (0.9%)
After four days	2 (1.8%)
Events related to head injury	
Road traffic accidents (RTA)	108 (97.3%)
Accidental falls at home	3 (2.7%)
External injuries on the head and neck	
Present	94 (84.7%)
Absent	17(15.3%)
GCS score	
GCS =13/14	49 (44.1%)
GCS=15	62 (55.9%)
CT-brain findings	
Positive (abnormal)	50 (45%)
Negative (normal)	61 (55%)

The data indicates that young males under 30 years of age, 33.3% (n=37), were predominantly involved in road traffic accidents, which made up most of the head injury cases in this study. Road traffic accidents (RTA) were responsible for 97.3% (n=108) of the cases. Approximately 45% (n=50) of the CT scans showed positive findings for head injuries despite the generally mild GCS scores. This highlights the critical role of neuroimaging, particularly due to the potential obscuring effect of alcohol intoxication on clinical assessment. The findings underscore the need for thorough evaluation and possibly routine CT imaging in similar scenarios to prevent overlooking significant injuries. The promptness of admission, with 87.4% (n=97) of patients being admitted on the same day, with an additional 1.8% (n=2) requiring admission after four days, emphasizes the necessity for vigilant monitoring and robust assessment protocols in emergency and trauma care settings.

Table 2 presents data analyzed using the Chi-squared test (Chi-squared value of 7.08) for independence to determine the significant association between two categorical variables: the Glasgow Coma Scale (GCS) scores and CT scan findings (abnormal vs. normal). The results show a significant p-value (0.012) for GCS scores of 13/14 suggesting that patients with these scores are more likely to have abnormal CT findings compared to those with GCS scores of 15. Thus, the result underscores the importance of considering GCS scores when interpreting CT scan results, as lower GCS scores are associated with a higher likelihood of detecting abnormalities on CT scans.

**Table 2:** Distribution of GCS and CT-brain findings among alcohol-intoxicated mild TBI patients Data are expressed as n and %. Fisher's exact test was used to compare the frequency between the groups. *indicates p<0.05 and is considered statistically significant. GCS - Glasgow Coma Scale; TBI - traumatic brain injury

GCS score	Abnormal CT findings, n=50 (45%)		Normal CT findings, n=61 (55%)		df	p-value
	n	%	n	%		
GCS= 13, 14	29	58	20	32.8	1	0.012*
GCS =15	21	42	41	67.2		

In Table 3 we compare CT-brain findings with the severity of alcohol intoxication. Out of 50 patients (45%) with positive CT-Brain findings, 41 patients (36.93%) were found to have objective scores of 6 or more. The chi-squared value was 2.15, and the overall p-value was non-significant at 0.433.

**Table 3:** Distribution of CT-Brain findings and the severity of alcohol intoxication in mild TBI Data expressed in n and %. The percentages represent the proportion of patients within each score range relative to the total number of patients in the study. *indicates p value <0.05 and is considered statistically significant. TBI - traumatic brain injury; NS - not significant

CT brain findings	No. of Patients with score 0-2 (mild), n=18 (16.21%)	p-value	No. of patients with score 3-5 (moderate), n=45 (40.53%)	p-value	No. of patients with definite score ≥6 (severe), n=48 (43.23%)	p-value
Normal study	16 (14.41%)	0.001*	38 (34.23%)	0*	7 (6.30%)	0.08 (NS)
Abnormal findings	02 (1.8%)		7 (6.30%)		41 (36.93%)	

For the 50 patients (45%) with positive CT findings suggestive of head injury, a retrospective evaluation was conducted to determine how many met the criteria for early CT-Brain imaging according to the Canadian CT Head Rule, NEXUS-II, and New Orleans Criteria. This is presented in Table 4.

**Table 4:** Comparison of the accuracy of the NEXUS-II head CT Rule, Canadian CT-head rule and the New Orleans Criteria retrospectively n represents the number of cases where the criterion was present. Sensitivity % (95% CI) indicates the proportion of true positives correctly identified by the criterion, along with the 95% confidence interval. NEXUS-II - National Emergency X-Ray Utilization Study II

NEXUS-II Head CT Rule		
Criteria	n/50	Sensitivity % (95%CI)
Skull fracture or hematoma	10	20 (9-31)
Loss of consciousness	18	36 (22-49)
Abnormal mental status	29	58(44-71)
Abnormal behavior	20	40 (26-53)
Age ≥65 years	7	14 (4-23)
Persistent vomiting	0	0
Neurologic deficit	0	0
Coagulopathy	0	0
Overall	36/50	72 (59-84)
Canadian CT Head Rule		
Amnesia	18	36 (22-49)
GCS <15 at 2 hours	36	72 (59-84)
Age ≥65 years	7	14 (4-23)
Open or depressed skull fracture	0	0
Signs of basal skull fracture	0	0
Vomiting ≥2 occurrences	0	0
Dangerous mechanism	9	18 (7-28)
Overall	43/50	86 (76-95)
New Orleans Criteria		
Headache	50	100 (93-100)
Amnesia	18	36 (22-49)
Age ≥60 years	10	20 (9-31)
Vomiting	1	2 (0.5-10)
Seizure	0	0
Intoxication	48	96 (86-99)
Signs of trauma above the clavicle	50	100 (93-100)
Overall	50/50	100 (93-100)

**Figure 2:**
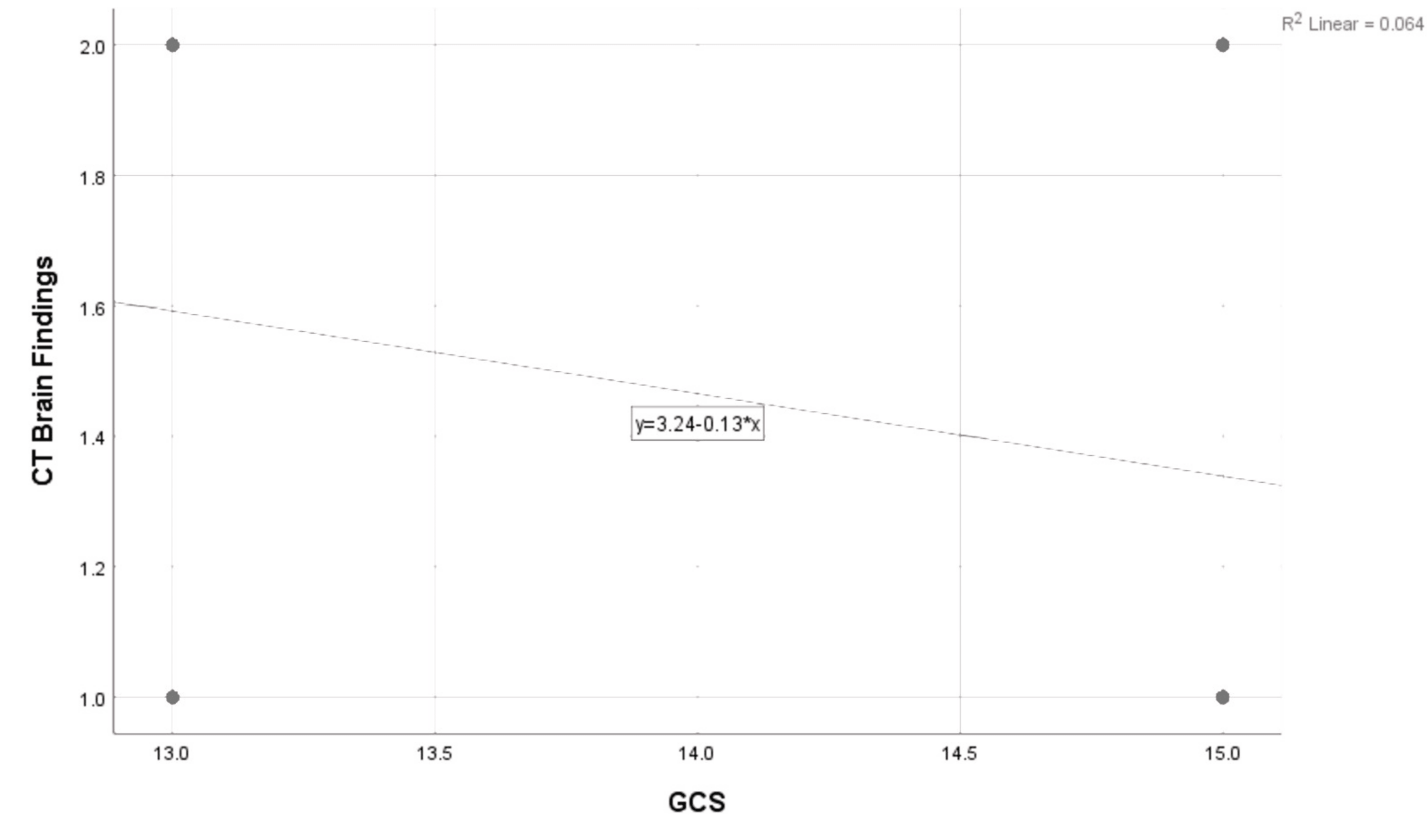
Correlation between GCS score and CT findings GCS - Glasgow Coma Scale

By using Pearson's correlation coefficient formula, a negative correlation (r=- 0.253) was found between GCS scores and CT-brain findings, as shown in Figure 2.

## Discussion

The results indicate that there is a statistically significant relationship between GCS scores and CT findings in this patient population, which aligns with the study's objective to assess the necessity of early CT scans in these cases.

Traumatic brain injury (TBI) is a major source of health loss and disability worldwide. Globally, the annual incidence of TBI is variably estimated at 64 to 74 million [8]. Around 75% of TBIs are mild and known as concussions. Most of the brain damage happens following the primary insult rather than at the time of the initial impact, and a complex biological sequence commences immediately after the trauma and can persist for hours to weeks thereafter. It is this secondary injury that considerably augments the overall morbidity and mortality associated with a traumatic brain injury [9]. Of all types of injury, those to the brain are the most likely to result in death or permanent disability [10]. Alcohol consumption has emerged as a prominent risk factor for traumatic brain injury due to factors such as the growing accessibility of alcohol, affordability, and the lack of well-defined regulations [10]. With an estimated 70 million users, India is confronting a significant burden of alcohol-related issues. The mortality and disability rates among those sustaining TBI under the influence of alcohol are nearly 1.5 to two times more than those without intoxication [11].

Weil et al. assessed alcohol abuse following traumatic brain injury and concluded that alcohol abuse following traumatic brain injury (TBI) is associated with poorer rehabilitation outcomes and a greatly increased chance of suffering future head trauma [12]. Our demographic data (Table 1) showed that young adults (below 30 years) were the most affected age group, which is consistent with the literature indicating that young adults are more likely to be involved in road traffic accidents (RTAs) and accidental falls [1,3]. The finding that 100% of the patients were male underscores the higher risk-taking behaviors and higher incidence of RTAs among males, as documented in previous studies [3, 4]. A significant portion of the patients (87.4%, n=97) were admitted on the same day of the injury, reflecting the acute nature of these incidents and the urgency in management. The predominant cause of head injury was RTAs (97.3%, n=108), which is in line with global data where RTAs are a leading cause of TBIs. External injuries in the head and neck region were present in 84.7% (n=94) of the patients.

Problems with GCS and alcohol intoxication

Though GCS remains the main instrument for classifying the severity of TBI as mild (GCS 13-15 with LOC), moderate (GCS 9-12), or severe (GCS ≤8), it, however, does not capture the specific patho-anatomical features or pathophysiology in individual patients, and is confounded by factors such as drug and alcohol use, medications, and tracheal intubation [13]. Shahin et al. assessed 188 patients with a head injury, comparing the GCS score at the time of admission and after 24 hours in both intoxicated and non-intoxicated groups; it was found that the GCS score improved significantly in the intoxicated group than the non-intoxicated group (p-value<0.001) [14]. In another study, in the patient group being under the influence of alcohol, mild and moderate head injuries were more common [15]. These findings might suggest that influenced patients can indeed get lower GCS scores. Similar results were replicated by Uccella et al., where the GCS score is overestimated in acutely intoxicated head trauma patients, and mild TBI could be safely managed by watchful waiting [16]. Another study has pointed out that GCS scores will remain largely unaffected even when blood alcohol levels (BAL) are greater than 200 mg/dl, but GCS over-estimated the severity of head injury in patients with intracranial bleeding and BAL above 200 mg/dl [17]. A study by Cook et al., which studied 107 patients, was unable to find a clear correlation between neurological scoring systems like GCS upon admission and one hour later and the presence of positive findings on CT-brain [18]. The above studies clearly show the inconsistencies between an objective bedside neurological examination like the GCS and positive CT-Brain findings in mild TBI under alcohol influence, calling into question the reliability of GCS scoring upon admission and planning further investigations and management in this cohort of patients.

In our study, the distribution of GCS scores revealed that 55.9% (n=62) of the patients had a GCS score of 15. However, the CT findings showed that 45% (n=50) of the patients had abnormal results, suggesting that even patients with a full GCS score might have significant intracranial injuries detectable on CT-brain. This emphasizes the importance of early CT imaging in alcohol-intoxicated patients with mild head injuries, as clinical evaluation alone might not be sufficient to rule out serious conditions.

Table 2 depicts the comparison between GCS scores and CT findings. The results show that a significant number of patients with lower GCS scores of 13-14 (44.1%, n=49) had more abnormal CT findings (58%, n=29), while those with a GCS score of 15 (55.9%, n=62) had predominantly normal CT results (67.2%, n=41). This suggests that lower GCS scores, contributed by alcohol intoxication, even within the mild range, are associated with a higher likelihood of abnormal CT findings, supporting the use of early CT imaging in these patients. So, it is upon the examining physician to decide if a CT-Brain is warranted in this subset of patients even if they do not fulfill the recommendations of the criteria set out by NICE (National Institute for Health and Care Excellence), UK [19].

Selective CT-scanning in mild TBI

The Canadian CT Head Rule (CCHR) and New Orleans Criteria (NOC) are developed clinical decision rules to guide CT use for patients with minor head injury and with Glasgow Coma Scale (GCS) scores of 13 to 15 for the CCHR and a score of 15 for the NOC [20]. In our study, we have shown the distribution of fulfilling criteria of different CT-Brain rules in mild TBI with varying levels of sensitivity in Table 4. The New Orleans Criteria showed the highest sensitivity (100%, 95% CI=93 to 100%, n=50/50) for abnormal CT findings, followed by the Canadian CT Head Rule (86%, 95% CI=76 to 95%, n=43/50) and the NEXUS-II Head CT Rule (72%, 95% CI=59 to 84%, n=36/50). Headache and external signs of injury over the clavicle had the highest association with positive CT-Brain findings observed in 100% of patients (n=50). These variations underscore the need for careful clinical judgment and possibly a combination of these criteria to guide CT scan decisions.

For clinically significant brain trauma, although less sensitive than the initial studies, the Canadian CT Head Rule and New Orleans Criteria rules successfully identified all patients requiring neurosurgical intervention. In contrast, the NEXUS-II rule exhibited the greatest potential reduction in CT scan utilization compared to other guidelines, but it failed to recognize some patients who underwent neurosurgery from its original study population [21].

Is early CT-brain necessary?

A study of mild TBI patients under alcohol intoxication showed conflicting results, with GCS 15 patients showing a higher percentage of positive CT-Brain findings than GCS 14 patients [16]. In our study, there was a negative correlation (r=-0.253) found between GCS scores and CT findings in Figure 2, indicating that higher GCS scores are slightly associated with fewer abnormal CT findings, although this relationship is very weak. Though one study showed that 90-95% of scanned patients with mild TBI do not exhibit intracranial injuries and risk exposure to radiation [13], considering the proportion of patients with positive CT-Brain findings in this study, we believe an early CT-brain is justified in this group, bearing in mind the significant long-term morbidity in undiagnosed and overlooked cases where immediate diagnosis and expert care would considerably reduce irreversible secondary brain injury and its sequelae. In the same study, elevated levels of biomarkers (still in research) in the blood of individuals with normal CT brain scans indicate the presence of underlying structural brain abnormalities, which are subsequently detected in up to 30% of mild TBI patients through MRI-brain.

Other studies have revealed the prevalence of clinically important injury in patients with minor head injury with acute alcohol intoxication to be significant and the necessity for early CT-Brain in this setting [22,23]. Also, early CT-brain can reliably identify patients with minor head injuries who can be safely discharged from the emergency department without hospitalization, even in the absence of a responsible caregiver. This approach can help avoid unnecessary hospital admissions for over 80% of mild TBI, thereby more effectively utilizing limited healthcare resources [23].

In this study, alcohol intoxication scores were also found to be highest (>6) in patients with positive findings on CT-brain (36.93%, n=41), as shown in Table 3, highlighting that those with a higher degree of intoxication were more disposed to head injuries, although the overall p-value at 0.433 was non-significant. Thus, a subnormal GCS at presentation, the history of consumption of alcohol, findings of external injuries on the head and neck, and history of loss of consciousness following the incident should prompt a CT-brain so as not to miss head injuries which may adversely affect patient's the quality of life, have long-term complications or potentially prove fatal. Three studies have shown that alcohol intoxication does not significantly reduce the GCS, and attributing lower GCS scores to intoxication would delay management [24-26]. Improvements in trauma care with low-cost solutions based on minimizing the time interval between injury and care, triage, and efficient hospital management can help to further reduce the serious outcomes of TBI [27].

Overall, the importance of early CT imaging in alcohol-intoxicated patients with mild head injuries to identify potential intracranial lesions that might not be apparent through clinical assessment alone is highlighted in the study. The findings support the use of objective criteria and clinical judgment in deciding the need for CT scans, ensuring timely and appropriate management to prevent long-term morbidity and mortality. This study contributes valuable data to the limited existing literature on the correlation between GCS scores and CT findings in alcohol-intoxicated mild head injury patients, particularly in a tertiary care setting.

Limitations

The smaller sample size is a significant limitation, restricting the ability to generalize outcomes to other mechanisms of trauma under acute alcohol intoxication. A larger sample size is needed to validate the accuracy of pre-CT assessment tools like the New Orleans Criteria, Canadian CT Head Rule, and NEXUS-II Criteria. Additionally, the socio-economic status of the subjects was not precisely determined. This study aimed to balance the use of selective CT-brain scans at a subsidized cost in a government hospital with limited imaging facilities and the safe observation of patients from lower socio-economic groups.

## Conclusions

The results indicate that alcohol intoxication alone does not significantly reduce GCS scores. Our study suggests that mild head injuries can be easily overlooked as symptoms and signs of head injury can be attributed to alcohol consumption, necessitating early CT scans for these patients. The cost-benefit analysis of this approach remains unclear and requires more comprehensive studies, especially considering the low-income, daily-wage workers in government hospitals with varying imaging facilities, policies, and subsidies. Future studies should focus on developing clinical decision rules to identify intoxicated patients at low risk of head injury who do not require CT investigation. Additionally, further investigation is warranted into the factors influencing physician decision-making regarding imaging for patients with mild traumatic brain injury under acute alcohol intoxication.

## Appendices

**Table 5:** Glasgow Coma Scale Score Mild Head Injury- GCS of 13, 14, 15 with a history of loss of consciousness Moderate Head Injury- GCS from 9 to 12 Severe Head injury- GCS from 3 to 8

Behaviour	Response	Score
Eye Response	Spontaneously	4
	To verbal command	3
	To pain	2
	No eye opening	1
Verbal Response	Oriented	5
	Confused	4
	Inappropriate words	3
	Incomprehensible sounds	2
	No verbal response	1
Motor Response	Obeys commands	6
	Localizes pain	5
	Withdrawal from pain	4
	Flexion to pain	3
	Extension to pain	2
	No motor response	1

**Table 6:** Objective-based scoring tool to determine the severity of alcohol intoxication Mild intoxication: Score 0-2 Moderate intoxication: Score 3-5 Severe Intoxication: Score 6-10

S.No	Criteria	Score
1.	Smell of alcohol on the patient	1
2.	Slurred speech/Increased verbosity	2
3.	Abnormal gait/ataxia	1
4.	Confusion/excitement	1
5.	Amnesia	2
6.	Nystagmus	2
7.	Bloodshot eyes	1
	Total	10

**Table 7:** CT-Brain decision rules- NEXUS-II head CT rule, Canadian CT-Head rule & New Orleans Criteria If any of the above risk factors for any one of the above criteria are identified then a CT head should be obtained.

NEXUS-II Head CT Rule	Skull fracture or hematoma
	Loss of consciousness
	Abnormal mental status
	Abnormal behavior
	Age ≥65 years
	Persistent vomiting
	Neurologic deficit
	Coagulopathy
Canadian CT Head Rule	Amnesia
	GCS <15 at 2 hours
	Age ≥65 years
	Open or depressed skull fracture
	Signs of basal skull fracture
	Vomiting ≥2 occurrences
	Dangerous mechanism
New Orleans Criteria	Headache
	Amnesia
	Age ≥60 years
	Vomiting
	Seizure
	Intoxication
	Signs of trauma above the clavicle
